# Optofluidic analysis system for amplification-free, direct detection of Ebola infection

**DOI:** 10.1038/srep14494

**Published:** 2015-09-25

**Authors:** H. Cai, J. W. Parks, T. A. Wall, M. A. Stott, A. Stambaugh, K. Alfson, A. Griffiths, R. A. Mathies, R. Carrion, J. L. Patterson, A. R. Hawkins, H. Schmidt

**Affiliations:** 1 School of Engineering, University of California Santa Cruz, 1156 High Street, Santa Cruz, CA 95064 USA; 2 ECEn Department, 459 Clyde Building, Brigham Young University, Provo, UT 84602 USA; 3 Department of Virology and Immunology, Texas Biomedical Research Institute, 7620 NW Loop 410, San Antonio, TX 78227 USA; 4 Department of Chemistry, University of California Berkeley, Berkeley, CA 94720 USA

## Abstract

The massive outbreak of highly lethal Ebola hemorrhagic fever in West Africa illustrates the urgent need for diagnostic instruments that can identify and quantify infections rapidly, accurately, and with low complexity. Here, we report on-chip sample preparation, amplification-free detection and quantification of Ebola virus on clinical samples using hybrid optofluidic integration. Sample preparation and target preconcentration are implemented on a PDMS-based microfluidic chip (automaton), followed by single nucleic acid fluorescence detection in liquid-core optical waveguides on a silicon chip in under ten minutes. We demonstrate excellent specificity, a limit of detection of 0.2 pfu/mL and a dynamic range of thirteen orders of magnitude, far outperforming other amplification-free methods. This chip-scale approach and reduced complexity compared to gold standard RT-PCR methods is ideal for portable instruments that can provide immediate diagnosis and continued monitoring of infectious diseases at the point-of-care.

Infectious diseases remain one of the main threats to human lives. Lower respiratory diseases and HIV/AIDS, for example, account for over 8% of all deaths worldwide^1^. Even in the case of non-fatal outcomes, infectious diseases cause significant societal costs via loss of productivity and health care expenses. The recent Ebola virus outbreak in West Africa^2^,^3^ serves as a powerful reminder that infectious diseases can be almost impossible to contain in today’s highly globalized world. The outbreak also brought into focus the need for early and differentiated diagnosis either before patients are visibly ill, or while symptoms such as fevers and headaches are nonspecific. In addition to being sensitive and accurate, the ideal diagnostic test should be reliable, rapid, have a wide dynamic range, use as little sample volume as possible, and be so easy to use that it can be deployed at the point of treatment. Existing nucleic acid tests fulfill many of these requirements. Real-time polymerase chain reaction (RT-PCR), which reverse transcribes, then amplifies small amounts of target genomic material followed by optical readout, is the gold standard test for hemorrhagic fevers as well as other infectious diseases such as influenza^4^,^5^,^6^. PCR boasts exquisite sensitivity and specificity, but is a complex technique, making it non-ideal for rapid point-of-use or point-of-care detection. Amplification-free nucleic acid detection is the obvious alternative, but other sensing approaches based on surface plasmon resonances, electrochemical, piezoelectric or advanced microscopy modalities have their own limitations [7,8 and references therein] and have not been able to replace PCR techniques.

Lab-on-chip based approaches have been considered as promising next generation techniques for medical diagnostics for at least two decades^9^. They enable instruments with compact footprints and require only small sample volumes. Recently, optofluidic approaches, that integrate both the biological sample handling and the optical readout in a single, chip-scale system, have received increased attention^10^,^11^,^12^. Such devices can lead to further miniaturization and simplification of a diagnostic instrument, add on-chip sample preparation capabilities^13^,^14^,^15^,^16^ and improve the detection sensitivity to the point where nucleic acid amplification is no longer needed.

Here, we report on the differentiated detection of Ebola virus using amplification-free, direct detection of single nucleic acids on a hybrid optofluidic device. We demonstrate that this system has the requisite *sensitivity, dynamic range*, and *specificity* for a viable clinical assay. Sample preparation and optical detection were carried out in dedicated layers that were optimized for their respective functions. By implementing a preconcentration step in the sample processing layer, we demonstrate single target detection over more than seven orders of magnitude and down to 0.2 pfu/mL, covering the entire clinically relevant concentration range^4^. The assay shows exquisite discrimination against other hemorrhagic fevers and points the way to a fully integrated front-to-back, amplification-free detection system.

## Results

**Figure 1:**
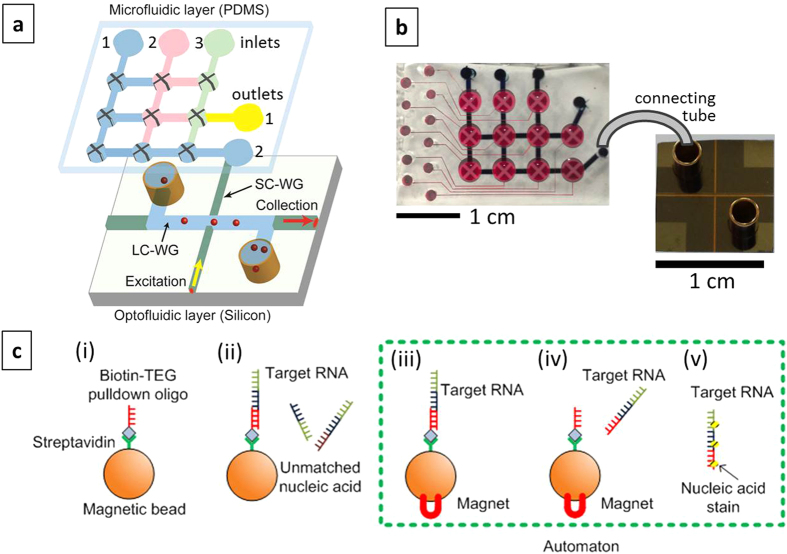
(**a**) Modular approach to hybrid optofluidic integration with individual chips dedicated to sample preparation and single nucleic acid detection; intersecting solid-core (SC-WG) and liquid-core (LC-WG) waveguides are highlighted in the optofluidic layer along with the planar optical beam geometry for single nucleic acid detection; (**b**) photographs of silicon-based optofluidic ARROW chip (bottom) and PDMS-based microfluidic automaton (top); pneumatic/sample channels are filled with red/blue dye for visualization of channel layers and layout; chips connect with flexible tubing; (**c**) Solid-phase extraction assay used for target isolation and detection. The dashed box indicates the steps that were implemented on the automaton chip.

Figure 1a shows our approach to creating an integrated device that is capable of carrying out both sample preparation and amplification-free detection. It consists of two layers (microfluidic and optofluidic) that are dedicated to different functions. This modular approach allows for separate optimization of often incompatible specifications^12^,^17^,^18^ and results in a flexible and reconfigurable system. The *sensitivity* required for amplification-free detection of single nucleic acids is provided by the optofluidic layer. Most chip-based detection approaches rely on specific binding of a target analyte to a surface to create a detectable optical or electronic readout^11^. In contrast, our approach is based on optical fluorescence detection of labeled targets flowing through a micro-channel that simultaneously acts as a liquid-core optical waveguide (LC-WG) as shown in the figure. A channel with 5 × 12 μm rectangular cross section is created using chemical vapor deposition and sacrificial layer etching on a silicon wafer (see Methods for fabrication details). Attachment of metal reservoirs (2 mm diameter) at the channel ends allows for controlled introduction of biological samples and pressure-based flow (see Fig. 1b, right, for a photograph of a completed chip). The reservoirs also serve as an interface with the microfluidic layer. Simultaneous guiding of light through liquids with low refractive index in this small channel is ensured by fabricating the hollow core on dielectric layers of SiO_2_ and Ta_2_O_5_ that reflect light via the antiresonant reflecting optical waveguide (ARROW) principle^19^,^20^. The sides and top of the channel are covered by a 6 μm thick SiO_2_ layer which can also be patterned into solid-core ridge waveguides to direct light to and from the liquid core. Single molecule detection sensitivity is enabled by the intersection of solid (SC) and liquid-core (LC) ARROWs at the center of the chip which defines a ~10 fL optical excitation/detection volume (see Methods). Fluorescence signals from molecules flowing past this point are collected by the liquid-core ARROW and routed to the edge of the chip as shown. This optically planar layout allows for straightforward integration of a second functional layer on top of the chip. Single particle detection of virions and microbeads with bound synthetic DNAs was previously demonstrated using purified targets in non-complex, non-clinical analytes^20^,^21^.

The need for integrated biological sample processing and large *dynamic range* is satisfied by the microfluidic layer, which consists of an array of lifting gate microvalves (automaton)^22^,^23^. This layer is created in PDMS (polydimethylsiloxane) using established soft lithography techniques (see Methods). Figure 1b (left) shows a photograph of an automaton, consisting of two sets of channels: a pneumatic layer for applying negative pressure to lift cross-shaped microvalves filled with red dye for visualization, and a fluidic layer (blue dye) in which sample analyte is transported between microvalves. The fluidic channels have much larger dimensions (130 × 800 μm) than the ARROW chip so they can quickly transport larger volumes (nL to mL) to and between the nine individual microvalve nodes on the chip, each holding 1.4 μL of liquid. Filling and interaction of these nodes is reconfigurable and software-controlled, allowing for flexible implementation of various sample preparation steps. The outlets can be connected to the ARROW chip with plastic tubing^18^, thereby creating a hybrid integrated optofluidic device. Finally, the *specificity* for accurately identifying different viral targets is created by the solid-phase extraction assay shown in Fig. 1c (see Methods for details). Briefly, magnetic microbeads are functionalized with a synthetic oligonucleotide designed to match the target (step (i)), in this case Ebola virus (EBOV). Targets bind to this pulldown sequence (step (ii)) and the beads are collected on a surface with a permanent magnet (step (iii)). Non-matching nucleic acids and other random biomolecules are then washed off and the remaining targets are thermally released from the beads (step (iv)), resuspended in buffer solution, and fluorescently labeled with SYBR Gold nucleic acid stain (step (v)). About 5 μL of this solution are then pumped through the ARROW chip for optical detection in under ten minutes. These preparation steps were carried out either entirely off-chip or on the automaton chip. The dashed box in Fig. 1c marks the steps that were implemented by the automaton in the present study.

**Figure 2:**
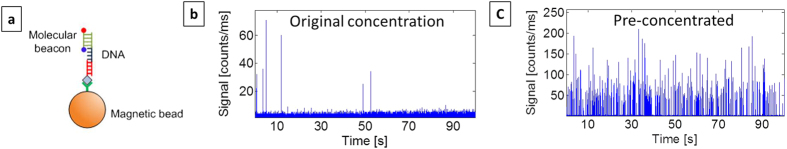
On-chip target preconcentration. (**a**) Particles to be concentrated: target oligomers are bound to pull-down recognition sequence on magnetic microbeads; molecular beacons specifically bind to target and cause beads to fluoresce; (**b**) particle fluorescence counts detected on ARROW chip before preconcentration step and (**c**) after preconcentration on automaton. A 335x concentration increase is observed.

In addition, the automaton is also capable of facilitating a pre-concentration step that allows for extending the detection limit of the ARROW sensor to presymptomatic target concentrations below 1 pfu/mL. For an initial demonstration and characterization of this feature, we used 100-mer synthetic oligonucleotide targets, corresponding to the protein coding region of the Ebola virus. These were mixed off-chip with magnetic beads and molecular beacons, resulting in a solution containing magnetic beads with hundreds of fluorescently labeled targets as shown in Fig. 2a. After diluting the beads in 3 mL of buffer solution, this analyte was pumped through the ARROW chip by applying negative pressure to the outlet reservoir, enabling optical detection, either directly or after preconcentration on the automaton. During preconcentration, a permanent magnet was placed under one of the microvalves as the analyte was pumped over it, continuously pulling down magnetic beads. Finally, the beads extracted from 3 mL of analyte were resuspended in ~5 μL of solution in outlet 1 (see Fig. 1) for transfer to the ARROW chip and optical fluorescence analysis. The unconcentrated solution (Fig. 2b) shows only 7 particles (magnetic beads with labeled target) over the course of the test (5 min total). After preconcentration, a constant stream of hundreds of particles is detected over the same period (Fig. 2c), demonstrating highly efficient on-chip concentration. Individual signal levels vary somewhat due to differences in the position of the particles within the waveguide cross section which results in variations in the excitation and collection efficiencies in accordance with the optical mode profiles in the solid-core excitation and liquid-core collection waveguide^21^. Variations due to microbead aggregation could be excluded by control observations from the top with a CCD camera. After correcting for the different flow speeds in each experiment, which results in different volumes tested during the identical assay time, a concentration factor of 335 was determined.

With these capabilities in hand, we carried out an assay to demonstrate amplification-free detection of Ebola virus using this integrated system. In the first set of experiments, all sample preparation steps (see Fig. 1c) were carried out off-chip. Supernatant of EBOV-infected Vero E6 cells was collected five days post infection. After inactivation, total RNA content was isolated for testing at a concentration of 2.1 × 10^5^ pfu/mL. Equivalent negative control samples of Sudan virus (SUDV, 2.2 × 10^5^ pfu/mL) and Lake Victoria Marburg virus (MARV, 4.4 × 10^6^ pfu/mL) were prepared. All concentrations were verified using PCR. In order to determine the dynamic range and limit of detection of our assay, these initial solutions were serially diluted, followed by the solid-phase extraction assay and the ARROW chip optical detection.

**Figure 3:**
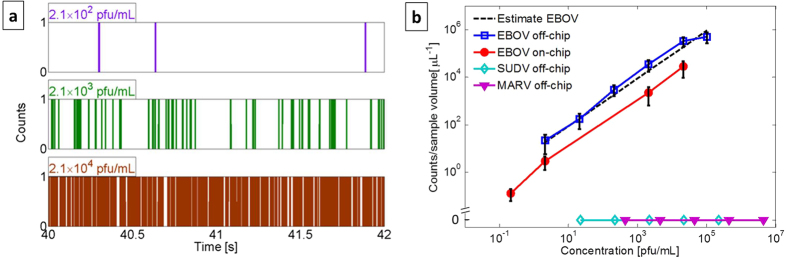
Amplification-free detection of Ebola virus on optofluidic chip. (**a**) Segments of digitized fluorescence counts above background showing concentration-dependent numbers of single RNAs; (**b**) concentration-dependent particle counts for off-chip (open squares) and using the automaton (solid circles) sample preparation. Negative controls (SUDV, MARV) did not create any counts (note the broken vertical scale). Dashed line: Predicted particle count determined from initial concentration and experimental parameters (tested sample volume, excitation and detection mode areas, liquid-core channel cross section). The lowest two concentrations for on-chip sample prep were reached by 50x and 460x preconcentration steps, respectively.

Single nucleic acids are identified by individual fluorescence peaks extending above a background level caused by free SYBR Gold dye. The detection threshold is defined as the maximum background level observed during a reference measurement with a dye-only solution of identical concentration immediately preceding the sample testing. Each signal exceeding this threshold is identified as an event and digitized, much like in single-photon counting. Figure 3a shows the resulting digitized signal traces for three different target concentrations (see Supplementary Information for original analog signals). Therefore, different concentrations are distinguished by the number of peaks per time as clearly seen in the figure. With single RNA sensitivity, any concentration can be detected as long as a reliable number of counts are collected. Therefore, the limit of detection is determined by the assay time (here between 3 and 10 minutes) which defines the amount of sample liquid tested and thus the maximum number of counts. Figure 3b shows the concentration dependent number of counts per starting volume of clinical sample. First, we find that both negative controls, SUDV and MARV, produce zero counts at all tested concentrations and flat lines in Fig. 3b (Note the broken scale on the ordinate axis to visualize zero counts on an otherwise logarithmic scale). The positive EBOV sample, on the other hand, creates positive counts with the expected concentration dependence. Here, we first consider the case of off-chip sample preparation (blue line). A linear concentration dependence is observed over 6 orders of magnitude down to 2.1 pfu/mL below which there are too few targets in our tested sample volume of a few microliters. The dashed line is an estimated count rate based on the initial number of target RNA copies independently measured by PCR. This concentration is converted into a count rate using the flow speed in the ARROW channel (measured by FCS analysis of each fluorescence signal) and the ratio of the liquid-core waveguide optical mode and the channel cross section area (only targets flowing within the mode area can be detected). We find excellent agreement between the expected and measured count rates within the experimental error (see Methods), confirming the ability of the ARROW chip to both identify and quantify a target virus in clinical sample material by direct nucleic acid counting.

In order to demonstrate a pathway towards complete on-chip diagnosis, we implemented sample preparation steps (iii–v) in Fig. 1c (target extraction, washing, and fluorescent labeling) on the automaton. In addition, we added a preconcentration step to further improve the limit of detection of this assay. The result for integrated sample processing and detection is shown by the red line in Fig. 3b. Again, we observe a linear dependence of the measured signal on concentration over six orders of magnitude. Moreover, by using 50x and 460x preconcentration (i.e. starting volumes of 0.25 mL and 2.3 mL, respectively) on the automaton chip, we were able to push the limit of detection to 0.2 pfu/mL, comparable with PCR analysis^5^, covering the entire range of viral loads observed in typical hemorrhagic fever cases^4^, and more than four orders of magnitude lower than other chip-based approaches^24^. The overall count rate for on-chip processing is lower than in the off-chip preparation case due to some nucleic acid and bead loss in the automaton channels. While this did not prevent us from improving the sensitivity of the assay, this issue can be mitigated by optimizing the automaton layout with shorter connecting channels and the surface treatment using a different coating^25^. The Supplementary Information shows the extension of the assay to the high-concentration, analog detection regime for a total continuous dynamic range of thirteen orders of magnitude.

## Discussion

In summary, we have demonstrated variant-specific identification and quantification of hemorrhagic fever infection in clinical samples using amplification-free counting of single nucleic acids. Both sample preparation and optical detection were implemented on-chip in a hybrid system with individually designed microfluidic and optofluidic layers. The system features excellent specificity, sensitivity and dynamic range. On-chip preconcentration was implemented to reach a limit of detection comparable to PCR. The demonstrated assay can be readily applied to other nucleic acid tests for infectious diseases or other applications. The high level of integration combined with reduced complexity makes this approach an attractive and user-friendly technology for point-of-use diagnosis, especially in resource-limited settings.

## Methods

### Optofluidic ARROW chip fabrication and waveguide structure

The optofluidic chip used in this study was fabricated on a 100 mm diameter silicon substrate on which a sequence of dielectric layers for optical guiding was deposited. These cladding layers consisted of Ta_2_O_5_ and SiO_2_ (refractive index: 2.107 and 1.47) having thicknesses in nm starting from the substrate of 265/102/265/102/265/102, where the material sequence reads SiO_2_/Ta_2_O_5_/SiO_2_/Ta_2_O_5_/SiO_2_/Ta_2_O_5_. SU8 photoresist (SU8-10, MicroChem) was spun on the wafer, patterned and developed to define the hollow waveguide channel with a rectangular cross section of 12 μm wide by 5 μm high. The hollow waveguide sits on a pedestal which was defined by dry etching in an inductively-coupled-plasma reactive ion etcher (ICP-RIE) using the SU8 and a thin nickel layer as the mask. A single SiO_2_ overcoat layer of 6 μm thickness was then deposited over the SU8 by plasma-enhanced chemical vapor deposition. Three micron tall ridges were etched into the SiO_2_ layer, again using the ICP-RIE, to form ridge waveguides that intersect multiple points of the hollow waveguide as illustrated in Fig. 1a. The excitation volume is determined by the optical mode of the ridge waveguide^26^. The Half Width Half Maxima (HWHM) of this excitation mode in horizontal and vertical directions are 1.6 μm and 1 μm, respectively. Fluid inlets into the hollow channel were exposed with a buffered HF wet etch through the top SiO_2_ layer and the SU8 was then removed with a H_2_SO_4_:H_2_O_2_ solution to form the hollow core. After rinsing the wafer in deionized water, it was cleaved into individual chips of approximately 10 × 12 mm^2^.

### Microfluidic (automaton) chip fabrication

Automata microfluidic devices were fabricated similar to previously described structures^18^,^22^,^23^. Lifting gate, or “actuate-to-open”, microvalves were created out of polydimethylsiloxane (PDMS) by forming a pneumatic layer that actuates a thin fluidic layer. Here, the device masters were created on silicon wafers using SU8-2100 photoresist which enabled creation of tall features (130 μm). When coupled with valve diameters of 3.75 mm, a single valve volume was 1.4 μL. In order to assemble the device, a 3 mm thick PDMS layer cast from the pneumatic master was bonded via oxygen plasma to a 200 μm thin PDMS membrane cast from the fluidic master. All inlets were created using a 2 mm biopsy punch (Miltex). The PDMS chip was then reversibly bonded to a glass slide via ozone treatment to create the final wall of the microfluidic channels.

### Pre-concentration demonstration on microfluidic automaton

In order to implement target pre-concentration on the microfluidic automaton, a test assay using synthetic nucleotide and molecular beacon labeling was performed. To this end, a 100-mer synthetic oligonucleotides corresponding to the protein coding region of Ebola Virus was used (EBOV, nt. 6832–6931), long enough to contain both a pulldown sequence for solid-phase extraction and a sequence for binding a fluorescent probe. Magnetic beads with a 50-mer pull-down sequence were used as the isolation media in this experiment (see also section on solid-phase extraction assay). As 50 of the 100 nucleic acids were targeted by the pull-down beads, the opposite end of the synthetic target was available for use in the fluorescence assay. A molecular hairpin beacon was designed to function as a specific tag for synthetic target nucleic acids: nt. EBOV 6900–6923 5′-dye-CGCATGGGCCTTCTGGGAAACTAAAAAATGCG-quencher -3′.

To begin the pre-concentration assay, equal concentrations of synthetic target and beacon (1 μM) were mixed and heated to 95 °C. In order to form thermodynamic products, the mixture was allowed to cool and slowly equilibrate to room temperature (~1 hour). The hybridized beacon-target complex was then added to 0.1 mg of pull-down beads and rotary mixed for 2 hours at room temperature. The automaton was flushed with a bovine serum albumin (BSA) solution prior to the experiment in order to minimize binding of beads and molecules to the PDMS surface. Then, the beacon-target-bead mixture was diluted to 3 mL and added to the automaton inlet. The pre-concentration process on the automaton is similar to the general automaton handling described below. However, after concentration of the solution into a single valve, the solution was not heated. Instead, the magnet was simply removed and the solution was pumped to the outlet (4.5 μL). The control assay consisted of pumping the above described bead solution (un-concentrated) directly through the automaton. This process accounts for wall binding and pumping losses and provides the base concentration of beacon-target-bead complexes.

### Filovirus Stocks, RNA Isolation, and preparation for fluorescence assay

Samples of three types of hemorrhagic fever viruses were prepared: Zaire Ebola virus from the Zaire Kikwit 1995 outbreak (GeneBank ID AY354458.1), Sudan Ebola virus strain Boniface (Genebank ID FJ968794.1) and Lake Victoria Marburg virus-Angola 2005 strain (Genebank ID DQ447660.1).

Stocks were used to infect T-175 tissue culture flasks containing Vero E6 cells at 90% confluence. The flasks were infected at a multiplicity of infection of 0.001, then incubated at 37 °C and 5% CO_2_ for 1 hour with gentle rocking. Dulbecco’s Modified Eagle Medium (DMEM) containing 2% fetal bovine serum was added and the flask was incubated until a 3+ cytopathic effect was observed. Supernatant was removed several days after infection (EBOV 5 days, SUDV 6 days, and MARV 5 days), clarified, and stored in 250-μL aliquots in an ultralow temperature freezer. Cyropreserved stocks were thawed and then extracted using Trizol LS reagents following manufacturer’s recommendations. Briefly, the sample was homogenized in a microfuge tube with Trizol LS reagent in a 3:1 ratio of Trizol LS volume to virus supernatant. Chloroform was added, mixed and then incubated at room temperature for 5 minutes. The tube was centrifuged at 12,000 × g for 15 minutes at 4 Degrees Celsius to separate the aqueous phase containing RNA. The aqueous phase was transferred to a new tube containing isopropanol, incubated for 10 minutes at room temperature, then centrifuged to precipitate RNA. Following a brief wash with ethanol, the pelleted RNA was resuspended in nuclease free water. The isolated RNA was then aliquoted and stored below −65°C until use. Virus stocks were also counted by conventional plaque titration according to standard methods^27^.

The concentration of virus particles was determined by a modified version of the loop drop method^28^,^29^. Briefly, virus was mixed thoroughly with polystyrene beads of known concentration (Duke Standards 3K/4K Series Particle Counter Standards). A small volume was dropped onto carbon-formvar coated 300-mesh grids and allowed to dry prior to fixation with 2% gluteraldehyde. Staining and sterilization occurred by exposure to 1% osmium tetroxide vapors for 1 hour and exposure to 1% uranyl acetate. Images were generated on a JEOL 100CX transmission electron microscope and enumeration of both virus particles and beads on the grid permitted determination of virus particle concentration. The measured titers were 2.1 × 10^5^ pfu/ml, 2.2 × 10^5^ pfu/ml and 4.4 × 10^6^ pfu/ml for EBOV, SUDV, and MARV, respectively. We also used PCR to determine the RNA concentrations: EBOV ~ 5.45 × 10^11^ copies/ml, SUDV ~ 1.08 × 10^10^ copies/ml and MARV ~ 1.38 × 10^11^ copies/ml.

For the fluorescence assay, serial dilutions of these samples were prepared in T50 buffer (10 mM Tris, 50 mM NaCl, pH 8.0): EBOV from 1.05 × 10^5^ pfu/ml to 0.21 pfu/ml, SUDV from 2.2 × 10^5^ pfu/ml to 22 pfu/ml, and MARV from 4.4 × 10^6^ pfu/ml to 440 pfu/ml.

### Solid-phase extraction assay for specific detection of Zaire Ebola virus

#### Magnetic bead preparation

A nucleic acid affinity media was used to isolated clinical samples. Dynabeads® MyOne™ Streptavidin T1 microspheres (Life Sciences) were coated in biotinylated pull-down oligonucleotide (IDT) following manufacturer specification. Pull-down beads were subjected to a 95 °C water bath for 5 minutes. This process ensured that any weakly bound streptavidin or nucleic acids would be removed prior to the experiment. The beads were then re-suspended in T50 buffer (10 mM Tris, 50 mM NaCl, pH 8.0) containing 0.1% Sodium Azide and were finally stored at 4 °C.

15-mer, 50-mer and 70-mer biotinylated pull-down sequences were chosen to be complementary to the protein coding region of EBOV (nt. 6832–6881 and 6832–6901, respectively). Furthermore, the region was chosen due to its sequence variability amongst hemorrhagic fever viruses. Particularly, SUDV and MARV (Genebank IDs FJ968794.1 and DQ447660.1) have no significant sequence alignments (BLASTn).

#### Sample preparation steps

Fig. 1c shows an overview of the sample preparation steps that were carried out both on and off chip to isolate and fluorescently label the target RNA. Steps *(i)* and *(ii)* show the preparation of magnetic beads with specific pull-down DNA sequence (pull-down beads) as described above.

First, the clinical total RNA samples prepared using the protocol described above were thawed and diluted to the desired concentration. They were then mixed with pull-down beads at a final concentration of ~1 × 10^9^ beads/ml for all samples at all concentrations. The mixed solution was heated up to 95 °C for 5 min and rotary incubated at room temperature for another 2 hours to enable sufficient binding between the target RNA (if present) and the pull-down oligomer (step (iii)). After the incubation, a magnet was used to pull down target bound beads, and random contaminating oligomers were washed away (step (iv)). After washing the magnetic beads twice, the magnetic bead solution was heated to ~80 °C to release the target RNA from the pull-down DNA (step (v)). A magnet was used again to pull down the beads and the supernatant containing target RNA (if present) was collected. The collected RNA solution was then mixed with nucleic acid stain (SYBR Gold) and incubated for another 20 min (step (v)).

When the sample preparation was carried out on the microfluidic automaton chip, on-chip nucleic acid pull-down, preconcentration, washing, releasing, and staining were performed in the following manner: first, mixed target clinical nucleic acid and pull-down microspheres were added to inlets 2 and 3 of the automaton, respectively (see Fig. 1a). A rare earth magnet mounted to a thermoelectric cooler (TEC) was moved into contact beneath an open valve (bottom row, 2^nd^ from left in Fig. 1b) of the automaton using a 3-axis micromanipulation stage, forming a magnetic incubation chamber. Actuation of the valves in peristaltic sequence moved solution from the mixture inlet to the magnetic incubation chamber in single valve volumes. Once the solution was suspended above the magnet, it was allowed to sit for 10 seconds in order to perform efficient magnetic pull-down of the microspheres. The remaining solution was pumped to a waste outlet and monitored for escaping beads via optical microscopy. This pumping/magnetic pull-down process was repeated until the mixture inlet was depleted of sample. As such, all of the magnetic beads from the inlet were concentrated into a single valve volume. Solution containing SYBR Gold dye (Life Sciences) was then pumped from automaton inlet 1 to fill the valves and incubation chamber with nucleic acid stain, while simultaneously rinsing away any contaminating materials. The magnet was removed and the beads were re-suspended in solution via flutter actuation of the incubation chamber’s valve. In place of the magnet, an equally sized, non-magnetic aluminum block was placed under the incubation chamber. The TEC was then set to 95 °C, yielding a measured temperature of ~80 °C on the upper surface of the automaton glass substrate. After releasing target nucleic acids for 10 minutes at this temperature, the aluminum block was replaced with the magnet and the beads were again pulled down. Solution from the SYBR Gold inlet was then used to drive the supernatant containing clinical nucleic acids from the incubation chamber to the outlet of the automaton. The sample was collected and input into the optofluidic ARROW chip for analysis.

### Amplification-free fluorescence detection of single nucleic acids on ARROW chip

The dye-labeled target RNA samples were pipeted into the liquid-core ARROW channel through one of the reservoirs connecting the inlet of the channel as shown in Fig. 1b. Negative pressure was applied to the other reservoir to enable constant flow inside the liquid-core ARROW channel. For fluorescence detection, an Ar-ion laser (488 nm) was coupled to the solid-core ARROW from a single mode fiber to excite the fluorescent RNA at the solid-core/liquid-core waveguide intersection. The generated fluorescence signal propagated orthogonally in the liquid-core ARROW channel (see Fig. 1a) and was guided to the chip edge where it was collected, filtered (Semrock, BLP01-532R-25) and detected by a photodiode (Perkin-Elmer, SPCM-AQR-14).

Each concentration was measured multiple times. Before each sample measurement, a control measurement using only SYBR gold dye was carried out to record the background fluorescent signal for the same amount of recording time (3 to 10 minutes) as the sample measurement. We selected the highest background photon count recorded in this way as the threshold for identifying a positive target detection event. The recorded photon counts for each event were normalized to the detected throughput power from the excitation waveguide output to eliminate the effect of power fluctuation due to fluctuations in fiber-to-chip coupling. For the negative controls (SUDV, MARV) no positive events were detected at any concentration. The positive target EBOV showed a strong concentration dependence as shown and discussed in Fig. 3 of the main text. Error bars represent the cumulative experimental uncertainty from multiple sources: variations in excitation volume due to fiber alignment, statistical variations of the starting concentrations (number of molecules in test volume given an average concentration), variations in pull-down efficiency, and target loss on the PDMS chip (for the on-chip preparation assay).

## Additional Information

**How to cite this article**: Cai, H. *et al*. Optofluidic analysis system for amplification-free, direct detection of Ebola infection. *Sci. Rep*. **5**, 14494; doi: 10.1038/srep14494 (2015).

## Supplementary Material

Supplementary Information
