# Supplementary material for: Optofluidic analysis system for amplification-free, direct detection of Ebola infection
Source: Sci Rep. 2015 Sep 25;5:14494. doi: 10.1038/srep14494 (PMC4585921; doi:10.1038/srep14494)
Supplement: Supplementary Information [file srep14494-s1.pdf]

Optofluidic analysis system for amplification-free, direct detection of Ebola infection

H. Cai[1*], J.W. Parks[1*], T.A. Wall[2], M.A. Stott[2], A. Stambaugh[1], K. Alfson[3], A. Griffiths[3], R.A. Mathies[4], R. Carrion Jr.[3], J.L. Patterson[3], A.R. Hawkins[2] and H. Schmidt[1a]

[1] School of Engineering, University of California Santa Cruz, 1156 High Street, Santa Cruz, CA 95064 USA.

[2] ECEn Department, 459 Clyde Building, Brigham Young University, Provo, UT 84602 USA.

[3] Department of Virology and Immunology, Texas Biomedical Research Institute, 7620 NW Loop 410, San Antonio, Texas 78227

[4] Department of Chemistry, University of California Berkeley, Berkeley, California 94720

[*]These authors contributed equally to the work.

[a] contact author. Email: hschmidt@soe.ucsc.edu, phone: 831-459-1482

SUPPLEMENTARY INFORMATION

Optimization of pull-down efficiency for solid-phase extraction

In order to investigate how the pull-down oligomer length affects the efficiency of the RNA selection, we prepared sets of magnetic beads with various length of pull-down DNA sequence: 15-mer (nt. 6832-6846), 50-mer (nt. 6832-6881) and 70-mer (nt. 6832-6901).

Fig. S1a shows the peak counts above background noise at various virus concentrations using 15-, 50- and 70-mer pull-down DNA sequences. It is immediately evident that longer pull-down sequences result in higher particle counts. Fig. S1b shows the peak counts above background noise at concentration of ~ 6.25×10^4 pfu/ml using 15-, 50- and 70-mer pull-down DNA sequences. The results show that longer pull-down DNA sequences provide better pull-down efficiency and thus give more peak counts in the measurement.

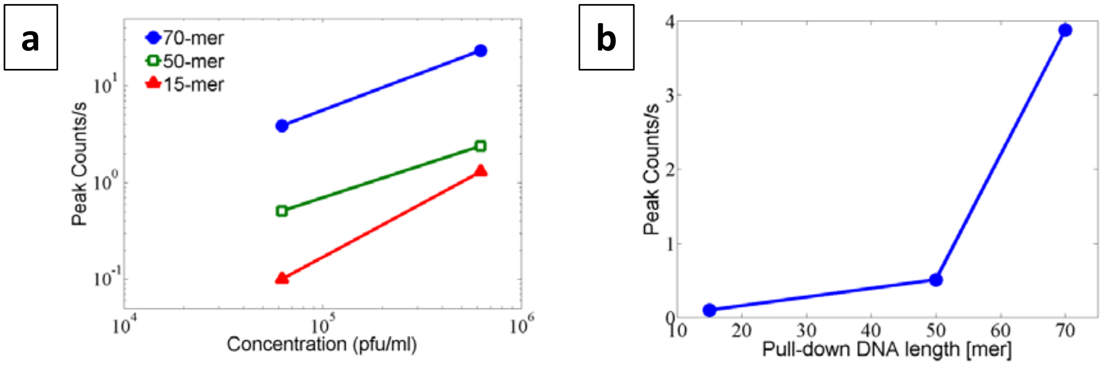

Fig. S1: **a**. Detected peak count upon various pull-down DNA lengths; **b**. Detected peak counts vs pulldown length at concentration of ~6.25×10^4 pfu/ml.

Fluorescence signals from individual viral nucleic acids

Fig. S2 shows the detected fluorescence signal above the background level for three concentrations of Ebola viral loads. An increasing number of peaks with concentration are observed. The peak height of each signal varies due to differences in the amount of bound dye and the position of the molecule within the waveguide cross section similar to Fig. 2b. These fluctuations on the single particle level have been previously analyzed by adding a nanopore which ensures single particle entry into the fluidic channel. They can be unambiguously assigned to individual particles [20,21]. For final quantitative analysis, each peak is digitized, i.e. assigned a normalized amplitude of one as shown in Fig. 3a in the main text.

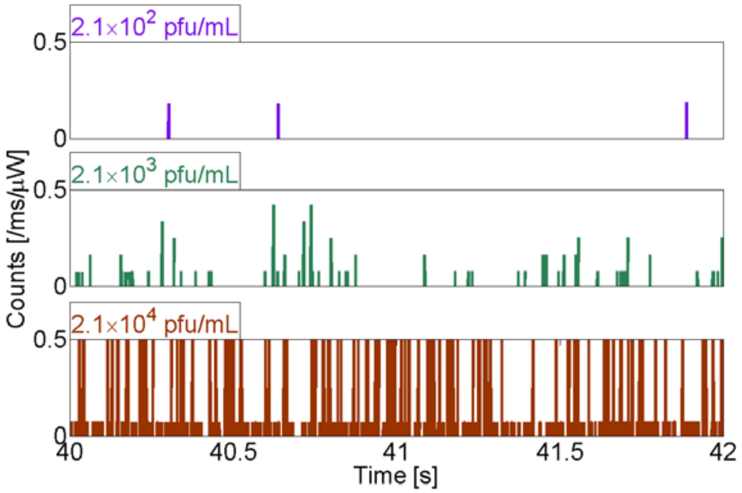

Fig. S2: Fluorescence signal above background for three concentrations of Ebola virus. Each peak is identified as detection of one particle regardless of absolute height and digitized for further analysis.

Analog detection of viral load in the high concentration limit

The main text focuses on detecting Ebola nucleic acid targets in the low (clinical) concentration limit with wide dynamic range. In order to demonstrate the full dynamic range of our chip-based detection, we carried out a series of experiments in the high concentration limit (more than 1 target on average in excitation volume) using a synthetic 100bp oligomer specific to Ebola virus H.sapiens-tc/COD/1995/Kikwit-9510621. We fluorescently labeled these oligomers off chip using SYBR gold nucleic acid stain. SYBR gold dyes nucleic acids ~1dye/4bp, resulting in ~25 fluorophores per target molecule. We prepared a serial dilution series of seven concentrations covering six orders of magnitude in concentration and measured the average fluorescence signal of each sample in an ARROW chip as shown in Fig. 1b in the main text. The results are shown in Fig. S3. We find a clear concentration dependence of the signal. The solid line is a linear fit, showing excellent linearity over the full concentration range. This demonstrates the ability for on-chip fluorescence detection of viral nucleic acids over six orders of magnitude in the high concentration limit and an overall dynamic range of at least 13 orders of magnitude.

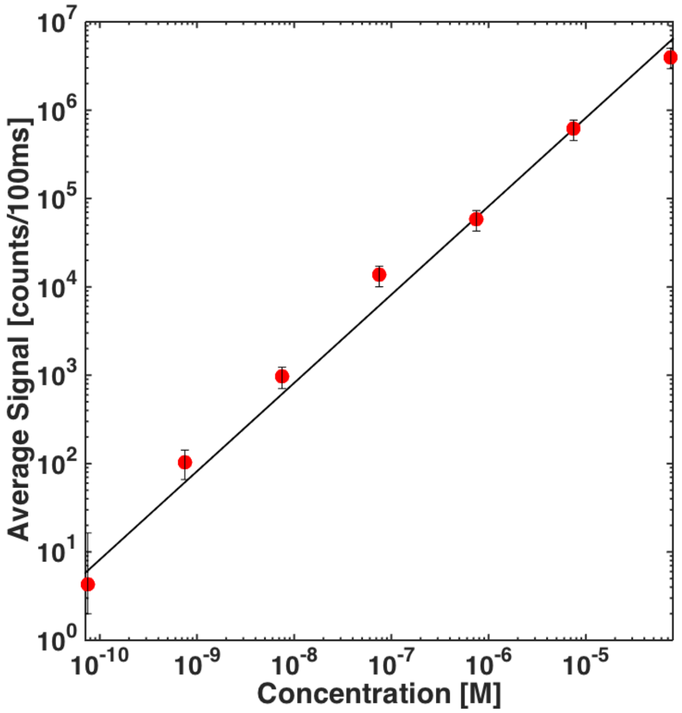

Fig. S3: Fluorescence concentration series in the analog detection regime demonstrating linear signal detection over six additional orders of magnitude (line: linear fit)
